# Aptamer-Based Biosensors for Point-of-Care Diagnostics

**DOI:** 10.3390/bios16010011

**Published:** 2025-12-23

**Authors:** Ana Díaz-Fernández

**Affiliations:** Instituto de Investigación Sanitaria del Principado de Asturias (ISPA), Avenida de Roma, 33011 Oviedo, Spain; anadfdez@gmail.com

## 1. Introduction

Over the past two decades, we have witnessed remarkable progress in the development of biosensors. What began as simple electrochemical devices in the early 20th century rapidly evolved to highly sophisticated diagnostic tools. Today, biosensors stand at the forefront of innovation, transforming healthcare, food safety, and environmental monitoring [1]. They are analytical devices that combine a biological recognition element with a physical transducer to detect specific analytes. Traditionally, antibodies and enzymes have played central roles in recognition, but recent advances have introduced aptamers, single-stranded synthetic DNA or RNA molecules, as a powerful new class of biorecognition agents. Aptamers offer several advantages over conventional biomolecules: they can be generated through in vitro selection, are chemically stable, easily modified, and can bind with high affinity to a wide range of targets, from small molecules to proteins and even whole cells [2].

At the same time, healthcare systems worldwide are shifting toward more accessible and decentralized testing. Point-of-care (POC) diagnostics, which enable rapid and accurate detection of disease markers directly at the patient’s side, are increasingly recognized as critical tools for improving clinical decision-making, reducing costs, and expanding access to underserved populations [3].

The integration of aptamers into biosensing platforms aligns perfectly with this vision: they allow for highly selective detection in compact, portable devices that can be tailored to diverse medical and environmental needs. By integrating aptamers into electrochemical, optical, and microfluidic platforms, researchers are developing compact devices capable of delivering precise diagnostic information outside of centralized laboratories. This innovation not only enhances accessibility to healthcare but also supports timely clinical decision-making in diverse settings, from hospitals to resource-limited environments [4].

In this context, the Special Issue “Aptamer-Based Biosensors for Point-of-Care Diagnostics” of *Biosensors* has collected nine works (three research articles and six reviews) that highlight the latest advancements and emerging trends in the field. Moreover, we successfully launched the second volume of this Special Issue following the success of the first volume.

## 2. Overview of Contributions

In this section, an overview and summary of the published articles are provided.

Alwarsh et al. (contribution 1) investigated how the size of silver nanoparticles (AgNP) and magnetic beads (MBs) can affect the generation of silver–gold galvanic exchange signals in magnetic electrochemical-based aptasensors. By comparing AgNPs of 20, 50, and 100 nm in size and MBs ranging from 100 to 4500 nm, the authors dissect how these physical parameters dictate electrochemical response, binding efficiency, and analytical sensitivity. They further contrast two conjugation strategies: traditional biotin-streptavidin linkage and an aptamer-mediated approach. The study reveals that intermediate dimensions, 50 nm AgNPs with 1000 nm MBs, strike the best balance between loading capacity, electrochemical accessibility, and signal reproducibility. Smaller AgNPs required higher concentrations to achieve similar silver content, while larger particles exhibited rapid signal saturation despite higher individual silver mass. Similarly, intermediate-sized MBs offered sufficient surface area for nanoparticle loading without imposing steric hindrance or obstructing electrode access, thereby supporting optimal electron transfer during assay readout. These insights provide practical design guidelines for aptamer-based electrochemical biosensors, informing future developments where nanomaterial dimensions are tuned to enhance signal transduction in point-of-care settings. Overall, this work advances our understanding of how nanoscale architecture affects biosensor performance and underscores the importance of rational design in developing next-generation diagnostics.

Asghar et al. (contribution 2) developed a fluorescence enzyme-linked aptamer assay (ELAA) that employs two different biotinylated aptamers as recognition elements in a sandwich format for the determination of creatine kinase-MB (CK-MB), a clinically important biomarker for acute myocardial infarction. After sandwich assembly, avidin-labeled alkaline phosphatase binds to the biotin-labeled aptamers, hydrolyzing its substrate, 2-phosphoascorbic acid trisodium salt, generating ascorbic acid. The enzymatically produced ascorbic acid acts as a reducing agent, inducing the structural deterioration of MoS_2_ nanosheets and their transformation. This transformation leads to the release of quenched aminated graphene quantum dots (AGQDs) and the restoration of fluorescence through Förster Resonance Energy Transfer (FRET) between the MoS_2_ nanoribbons and AGQDs. The coupling of enzyme amplification with nanomaterial-based FRET results in a turn-on fluorescent signal that correlates directly with CK-MB concentration. The sensor demonstrates a working detection range from 2.5 to 160 nM and a limit of detection of 0.20 nM, showcasing high sensitivity within clinically relevant ranges. Recovery experiments in spiked human serum further confirm the practical applicability of the aptasensor.

Wan et al. (contribution 3) presented a biosensor integrated with a reusable printed circuit board (PCB) and functionalized glucose test strips designed for rapid and non-invasive breast cancer screening through the detection of two key salivary biomarkers, HER2 and CA15-3. In this work, commercially available glucose test strips were functionalized with specific receptors and integrated into a custom-designed PCB containing a microprocessor, DAC, pulse generator, and closed-loop amplifier to enable precise signal generation and measurement. The authors systematically optimized operating parameters, including reference voltage, test voltage, and amplifier gain, to maximize analytical performance. Under these optimized conditions, the biosensor achieved a limit of detection of 10^−15^ g/mL for both biomarkers and rapid signal acquisition within approximately one second, representing a sensitivity 4 to 5 orders of magnitude greater than conventional ELISA. Moreover, the sensor was clinically validated using 29 human saliva samples, demonstrating clear distinctions between healthy individuals and patients with in situ or invasive breast cancer, underscoring the method’s diagnostic capability. The incorporation of Bluetooth Low-Energy Communication (BLE) enables remote monitoring, reduces hospital visits, and enhances accessibility for point-of-care and mobile screening applications.

Lobo-Castañón et al.’s (contribution 4) work provided a roadmap for future research and interdisciplinary collaboration in precision oncology by outlining the technological and translational trajectory of aptamer-based CRC diagnostics. Specifically, they examined established and emerging CRC biomarkers and discussed recent advances in aptamer selection and design, with a focus on SELEX variants and in silico optimization approaches tailored to CRC-relevant targets. Moreover, they reviewed the published works integrating aptamers into cutting-edge sensing platforms, such as electrochemical, optical, and nanomaterial-enhanced aptasensors, with emphasis on recent innovations that enhance sensitivity, portability, and multiplexing capabilities. Furthermore, they explored the convergence of aptasensing with microfluidics and wearable technologies to enable intelligent, miniaturized diagnostic systems. Finally, they considered clinical and regulatory pathways for point-of-care implementation, as well as current challenges and opportunities for advancing the field.

Wu et al. (contribution 5) summarized the basic principles of nucleic acid strand displacement reactions (SDRs) and recent advances in proximity activation strategies, emphasizing the role of strand proximity as a central driving force. They classified SDR methods according to their nucleation principle: canonical toehold-based reactions represent proximity activation through base-pairing, while non-canonical strategies rely on alternative molecular interactions, with particular emphasis on how aptamers can be employed to activate SDRs. They further discussed how combining different activation and kinetic control approaches gives rise to dynamic networks with complex and dissipative behaviors, offering new directions for DNA-based nanotechnology and aptasensors.

Zhou et al. (contribution 6) explored the fundamental principles of CRISPR-Cas systems, focusing on various Cas proteins (Cas9, Cas12a, Cas13a) and their distinct mechanisms of action (cis- and trans-cleavage). The work highlighted diverse applications such as infectious disease surveillance, cancer biomarker detection, and genetic disorder screening, emphasizing key advantages like speed, high sensitivity, specificity, portability, and cost-effectiveness, particularly for POC testing. It also discussed current challenges, including sensitivity limitations without pre-amplification, specificity issues, and complex sample preparation, while outlining promising future directions such as artificial intelligence (AI) integration and the development of universal diagnostic platforms to enhance clinical translation.

Khalid-Salako et al. (contribution 7) provided a comprehensive review of the latest developments in surface plasmon resonance (SPR)-based aptasensors. The review discussed key principles of SPR, strategies for immobilizing aptamers on sensor surfaces, and design approaches to enhance sensitivity and selectivity. It highlighted diverse applications in medical diagnostics, drug discovery, and environmental monitoring, while also addressing challenges such as reproducibility, real-sample testing, and translation to commercial devices. Overall, the article underscores the potential of SPR aptasensors to combine advanced optical detection with versatile molecular recognition for next-generation biosensing technologies.

Wang et al. (contribution 8) reviewed recent progress in the design and use of photoactivable aptamer biosensors for POC. Photoactivable biosensors use light-responsive mechanisms to control aptamer target recognition. In this work, they explained how integrating photoresponsive elements into aptamers allows precise spatiotemporal control of molecular binding, improving sensitivity, selectivity, and dynamic response, and discussed the various optical regulation strategies and materials used in these systems. The use of photoactivated biosensors enhances the performance of portable diagnostic platforms in detecting diverse targets in POC settings, while also addressing current challenges and future prospects for advancing precision diagnostics and environmental monitoring.

Mauriz et al. (contribution 9) systematically reviewed recent advances in SPR aptasensors for viral diagnostics by analyzing 29 studies identified according to Preferred Reporting Items for Systematic Reviews and Meta-Analyses (PRISMA) guidelines, focusing on how aptamer-based SPR platforms detect virus proteins or whole viruses and assessing their diagnostic performance via a meta-analysis. It showed that SPR aptasensors have promising sensitivity and specificity across a range of viruses, including influenza, SARS-CoV-2, and HIV, with conventional SPR configurations yielding the most consistent diagnostic results. However, despite their analytical capabilities, significant challenges remain for clinical implementation, such as the impact of complex biological samples, inconsistent reporting of diagnostic accuracy metrics, and technical barriers to robust, real-world use. The review concluded that advancing stable surface chemistries, integrating clinical samples, and improving standardized accuracy assessments are crucial for translating SPR aptasensors into reliable tools for virus detection beyond the laboratory.

## 3. Conclusions and Outlooks

While the promise of aptamer-based biosensors for point-of-care diagnostics is undeniable, several challenges remain before widespread clinical adoption can be realized. Key issues such as regulatory approval, large-scale manufacturing, integration with digital health platforms, and ensuring robustness in diverse real-world settings must be addressed. At the same time, advances in nanomaterials, microfluidics, and data analytics are opening new opportunities to enhance sensitivity, portability, and user-friendliness. The future of this field will depend on close collaboration between chemists, biologists, engineers, clinicians, and industry partners to translate laboratory innovations into accessible diagnostic tools. The contributions gathered in this Special Issue not only showcase the current state of the art but also will inspire the next generation of biosensor technologies that bring precision diagnostics directly to the patient’s side.
